# Premature atrial contractions: A predictor of atrial fibrillation and a relevant marker of atrial cardiomyopathy

**DOI:** 10.3389/fphys.2022.971691

**Published:** 2022-10-24

**Authors:** Jean-Baptiste Guichard, Eduard Guasch, Frederic Roche, Antoine Da Costa, Lluís Mont

**Affiliations:** ^1^ Arrhythmia Unit, Hospital Clínic de Barcelona, Institut d’Investigacions Biomèdiques August Pi iSunyer (IDIBAPS), Universitat de Barcelona, Barcelona, Spain; ^2^ Centro de Investigación Biomédica en Red Enfermedades Cardiovasculares (CIBERCV), Madrid, Spain; ^3^ Department of Cardiology, University Hospital of Saint-Étienne, Saint-Étienne, France; ^4^ Sainbiose, DVH, Inserm U1059, University Hospital of Saint-Étienne, Saint-Étienne, France

**Keywords:** premature atrial contraction, nonsustained atrial arrhythmia, atrial cardiomyopathy, atrial fibrillation, ischemic stroke

## Abstract

An increased burden of premature atrial contractions (PACs) has long been considered a benign phenomenon. However, strong evidence of their involvement in the occurrence of atrial fibrillation (AF), ischemic stroke, and excess mortality suggests the need for management. The central question to be resolved is whether increased ectopic atrial rhythm is only a predictor of AF or whether it is a marker of atrial cardiomyopathy and therefore of ischemic stroke. After reviewing the pathophysiology of PACs and its impact on patient prognosis, this mini-review proposes to 1) detail the physiological and clinical elements linking PACs and AF, 2) present the evidence in favor of supraventricular ectopic activity as a marker of cardiomyopathy, and 3) outline the current limitations of this concept and the potential future clinical implications.

## Introduction

Both premature atrial contractions (PACs) and nonsustained atrial firing are frequent conditions. Historically considered a benign phenomenon, challenging data have highlighted its impact on morbidity and mortality, whether in terms of atrial fibrillation (AF), ischemic stroke or all-cause and cardiovascular mortality (Himmelreich et al., 2019; Huang et al., 2020; Meng et al., 2020). However, the diagnostic and therapeutic management of ectopic atrial rhythm is not yet precisely standardized (Farinha et al., 2022). To improve its management, the central unresolved question is whether an increased PAC burden is only a predictor of AF or whether it is also a marker of atrial cardiomyopathy and subsequent ischemic stroke.

After reviewing the clinical features of PACs, the objective of this mini-review is to provide physiological and clinical data regarding the relationship between nonsustained atrial arrhythmia and AF on one hand and atrial cardiomyopathy on the other hand.

## Physiopathology and epidemiology of nonsustained atrial arrhythmia

**FIGURE 1:**
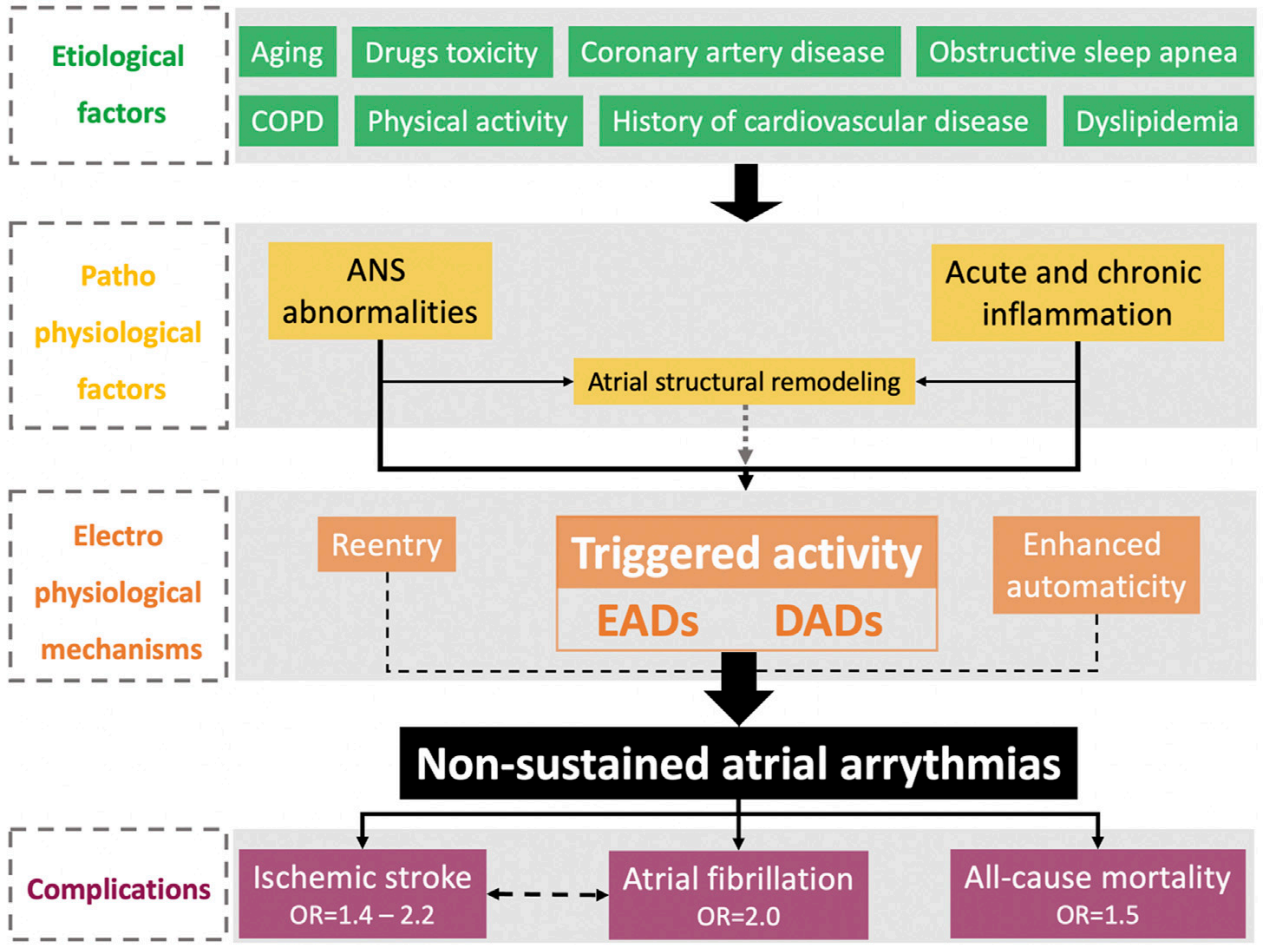
Physiopathological and epidemiological features of nonsustained atrial arrhythmias. ANS, autonomic nervous system; COPD, chronic obstructive pulmonary disease; DADs, delayed afterdepolarizations; EADs, early afterdepolarizations; OSA, obstructive sleep apnea.

The complex pathophysiology of PACs and their clinical implications for increased morbidity and mortality are summarized in Figure 1.

### Physiopathological of nonsustained atrial arrhythmias

PACs are secondary to triggered activities, either by early afterdepolarization in the case of prolongation of the action potential of atrial cardiomyocytes or by delayed afterdepolarization in the case of increased heart rate (Heijman et al., 2014). Calcium-handling abnormalities leading to intracellular calcium overload play a key role in the occurrence of triggered activity secondary to RyR2 dysfunction and increased calcium release from the sarcoplasmic reticulum, and uptake decreases secondary to the SERCA2a channel (Heijman et al., 2012). Atrial structural remodeling, including fibrosis, is mainly involved in conduction velocity abnormalities and re-entrant mechanisms leading to the initiation and maintenance of sustained arrhythmia. However, atrial structural remodeling is also involved in the occurrence of triggered activities by two distinct mechanisms. Abnormalities in calcium handling are increased in cases of significant structural remodeling (Burstein and Nattel, 2008; Choi et al., 2012), and afterdepolarizations can be initiated by direct cardiomyocyte-fibroblast interactions (Yue et al., 2011). Thus, nonsustained atrial arrhythmias are mainly promoted by electrical atrial remodeling but could also be driven by structural remodeling (Schotten et al., 2011).

Autonomic nervous system (ANS) abnormalities play a crucial role in the occurrence of supraventricular ectopic activity (Chen et al., 2014). At the biochemical level, sympathetic stimulation via *β* receptors is a trigger for calcium release from the sarcoplasmic reticulum (Shen et al., 2012). This could explain the increase in PAC burden during physical activity (Younis et al., 2018). Both the sympathetic and parasympathetic systems are involved in the occurrence of PACs by increasing the calcium transient and heterogeneously reducing action potentials. Atrial cavities are highly innervated by the ANS via the ganglionated plexi and the left atrial ridge, mainly at the level of Marshall’s vein (Lin et al., 2008). Ablation of the ANS connections leads to a drastic reduction in the PAC burden in an animal model (Lu et al., 2009). Global ANS breakdown is also implicated. PAC burden in a population with chronic autonomic failure is associated with decreased sympathetic and parasympathetic activity (Goldstein, 2010). In a large cohort study, PAC burden was associated with both ANS imbalance and decreased global activity (Huang et al., 2020). ANS abnormalities seem to be involved in PAC physiopathology through structural remodeling. Independent of blood pressure changes, renal denervation leads to a decrease in PAC burden (Schirmer et al., 2015) and LA dilation (McLellan et al., 2015).

Acute inflammation is a major risk factor for PAC, such as that occurring in the postoperative period (Jidéus et al., 2006; Dobrev et al., 2019). Chronic inflammation involving the enhancement of oxidative stress also seems to be involved in PAC physiopathology (Anumonwo et al., 2017; Shen et al., 2019).

### Epidemiology of nonsustained atrial arrhythmia

Different risk factors are associated with PAC occurrence (Kerola et al., 2019), such as aging (Tasaki et al., 2006; Conen et al., 2012), coronary artery disease (Zoni Berisso et al., 1986), obstructive sleep apnea (Rossi et al., 2013; Kawano et al., 2014), history of cardiovascular disease, heart failure, structural heart disease, physical activity, dyslipidemia, drugs (Conen et al., 2012), and chronic obstructive pulmonary disease (Kusunoki et al., 2016).

Epidemiologically, PACs are common in the general female (Sobotka et al., 1981) and male (Brodsky et al., 1977) populations. Only 1% of individuals in the general population do not present any PACs over a 24-h recording (Conen et al., 2012), and the PAC prevalence based on a 12-lead ECG is approximately 0.5% (Loomba et al., 2018).

Three recent meta-analyses assessed the association between PACs, morbidity, and mortality (Himmelreich et al., 2019; Huang et al., 2020; Meng et al., 2020). The presence of a significant PAC burden is associated with a 3-fold increase in AF occurrence and is independently associated with a 2-fold increase. Nonsustained atrial arrhythmia is also associated with all-cause mortality in both univariate (OR = 2.15) and multivariate (OR = 1.5) analyses. These results are conflicting since another study identified only PVC-associated PACs as a predictor of all-cause mortality (Cheriyath et al., 2011). Increased PAC burden is a predictor of ischemic stroke occurrence (OR between 2.20 and 2.54) and seems to be independently associated (adjusted OR between 1.41 and 2.23). This association was studied not only in the general population (Kamel et al., 2013) but also among patients exhibiting an ischemic stroke with an unknown origin after a systematic evaluation, i.e., an embolic stroke from an undetermined source (ESUS) (Qureshi et al., 2014). The association between increased PAC burden and potential morbidities is stronger in women (O’Neal et al., 2016a).

Nonsustained atrial arrhythmias are therefore associated with AF occurrence. However, there are two hypotheses to explain this association: either PACs are an epidemiological risk marker for AF, or there is a causal relationship between these two conditions.

## Nonsustained atrial arrhythmia as a predictor of atrial fibrillation

### Physiopathological relationship between premature atrial contractions and atrial fibrillation

The majority of supraventricular tachyarrhythmias, including AF, are triggered by single or consecutive PACs (Durrer et al., 1967), setting a functional line of conduction block (Waldo, 2002). The PAC site plays an important role in the initiation of AF. The pulmonary veins (PVs), as the preferential site of AF occurrence secondary to nonsustained atrial arrhythmias, were reported for the first time in 1998 (Haïssaguerre et al., 1998). Although one-half of the PACs registered in AF patients are located in extra-PV sites, both PACs from PVs and PACs with a bifocal origin are more susceptible to inducing AF (Schmitt et al., 2002). PV-related PACs are also involved in the initiation of atrial flutter (Schneider et al., 2015). A topographic mismatch between arrhythmogenic sites, such as PVs and the superior vena cava, and non-AF locations is steady-grounded (Wang et al., 2017).

Although the majority of AF episodes are preceded by an increase in atrial ectopic rhythm (Dimmer et al., 2003), only 40% of PACs are responsible for sustained arrhythmia in the AF population (Schmitt et al., 2002), and the PAC burden has a poor predictive capacity for AF initiation (Hnatkova et al., 1997). Several electrophysiological features are needed to initiate PAC-related AF. Although few cases of late-coupling PAC-induced AF have been reported (Kinoshita and Katoh, 2007), PAC-inducing AF has a short coupling interval (Capucci et al., 1992). Patients with PAC-induced AF face prolonged refractory periods after PAC, while PAC tends to shorten the dispersion of atrial refractory periods when patients are free of sustained arrhythmia (Chauvin and Brechenmacher, 1989). Finally, PACs may induce calcium-handling abnormalities and spatial and dynamic dispersion of repolarization duration among atrial cardiomyocytes, including discordant alternans, causing conduction block, re-entry, and AF initiation (Gong et al., 2007). The sites with the largest vulnerable window are located in the PVs, highlighting the link between electrophysiological features and preferential PAC sites.

Some modulators, such as ANS imbalance, play a crucial role in AF promotion (Scherlag et al., 2005). The occurrence of PACs in patients presenting variability in the PR interval leads to a higher AF risk (Chun et al., 2016). Enhancement of vagal tone seems to be a trigger of PAC-induced AF initiation: heart rate turbulence, a marker of vagal tone, is higher in pre-AF periods and is also associated with PAC burden (Vikman et al., 2005).

### Clinical evidence of premature atrial contraction-induced atrial fibrillation mechanism

The first invasive AF ablation was based on radiofrequency ablation of PVs located in the PVs and was proven to be an effective therapeutic strategy allowing a 62% rate of AF-free survival after a 1-year follow-up (Haïssaguerre et al., 1998). An increased PAC burden after AF ablation is associated with the failure of PV isolation assessed in a redo procedure. A redo PV isolation procedure leads to a drastic decrease in PAC and AF burden, suggesting the crucial role of PACs located in the PVs as triggers of AF (Yamane et al., 2006). Patients with AF recurrence after PV isolation had a lower PAC burden before ablation, suggesting the significant role of PACs from PVs in the proarrhythmic process (Hamon et al., 2021).

On the other hand, extra-PV PACs are a marker of atrial remodeling. Both PAC burden (Gang et al., 2015) and rapid atrial firing (Inoue et al., 2020) after successful PV isolation are predictors of AF recurrence during long-term follow-up. Thus, it seems that ectopic atrial rhythm is a marker of atrial substrate responsible for AF recurrence after PV isolation, although a failure of PV isolation cannot be ruled out.

PAC burden seems to be associated with long-term recurrence of AF after AF ablation (Egami et al., 2021) regardless of AF recurrence during the blanking period. Interestingly, a patient with a low PAC burden and early blanking period-related AF recurrence has a low risk of long-term AF recurrence (Alhede et al., 2018).

Consistent data suggest a physiopathological and clinical relationship between PACs and sustained supraventricular arrhythmia, leading to electrophysiological atrial remodeling. However, the concept of increased PAC burden as a marker of global atrial remodeling, so-called atrial cardiomyopathy, challenges the classic paradigm.

## Premature atrial contractions as a marker of atrial cardiomyopathy

### The concept of atrial cardiomyopathy

Atrial cardiomyopathy was precisely defined in 2017 by an expert consensus as any complex structural, architectural, contractile, or electrophysiological changes affecting the atria with the potential to produce clinically relevant manifestations (Goette et al., 2017). This new paradigm suggests that both AF and cardioembolic events are secondary to atrial cardiomyopathy and challenges the traditional concept of AF as directly inducing thromboembolic events (Guichard and Nattel, 2017). Emerging data suggest the lack of a direct link between AF and stroke. First, although the epidemiological relationship between AF and ischemic stroke is steady-grounded, there is a lack of clear temporal association between AF and stroke occurrence as assessed in the ASSERT (Healey et al., 2012) and TRENDS (Daoud et al., 2011) trials. Second, the thromboembolic risk (TER) is not homogeneous in the AF population and is highly influenced by the presence of cardiovascular risk factors (Lip et al., 2010). The CHA_2_DS_2_-VAS_c_ score is a clinical diagnostic tool allowing for TER assessment (Hindricks et al., 2020) and encompasses different risk factors for atrial cardiomyopathy, such as heart failure, hypertension, diabetes mellitus, vascular disease, and aging (Guichard and Nattel, 2017). Third, the benefit of anticoagulation secondary to subclinical AF screening on the TER is uncertain (Svendsen et al., 2021; Svennberg et al., 2021). Finally, early rhythm control in AF patients decreases ischemic stroke occurrence (Kirchhof et al., 2020), but the underlying mechanism is not understood. This finding could be explained by a decrease in AF burden or the prevention of AF-induced atrial cardiomyopathy.

A cardioembolic stroke could occur in the presence of atrial cardiomyopathy even before AF occurrence, as encountered in cases of cardiac amyloidosis (Dubrey et al., 1995). A diseased left atrium is characterized by inflammation (Kamel et al., 2016), endothelial dysfunction (Cai et al., 2002), fibrosis (Burstein and Nattel, 2008), contractile dysfunction (Mihm et al., 2001), and dilation (Vaziri et al., 1994). However, there is currently a lack of standardized and validated clinical assessment of atrial cardiomyopathy and a need for validation of clinical markers of atrial cardiomyopathy (Bisbal et al., 2020). Assessment of the PAC burden could be a relevant parameter to clinically characterize atrial cardiomyopathy (Farinha et al., 2022).

### Nonsustained atrial arrhythmias as a marker of thromboembolic risk

Supraventricular ectopic activity is an independent predictor of stroke occurrence (Huang et al., 2020) after adjustment for cardiovascular risk factors (Engström et al., 2000), ECG abnormalities (Murakoshi et al., 2015), ethnicity, and cardiovascular medication (O’Neal et al., 2016a). Furthermore, an increased PAC burden is associated with nonlacunar ischemia but not with all-cause stroke, suggesting a link between PACs and cardioembolic events (O’Neal et al., 2016a). Atrial firing is also an independent predictor of ischemic stroke recurrence in an ESUS population (Sejr et al., 2020).

Different mechanisms could underlie the relationship between ectopic atrial rhythm and ischemic stroke. PACs and ischemic stroke share the same risk factors, such as aging, heart failure (Conen et al., 2012), and OSA (Kawano et al., 2014). However, the PAC burden is still predictive of stroke occurrence even after a seemingly thorough adjustment for shared risk factors (Huang et al., 2020). PAC as a pre-AF status could highlight in an upstream manner the role of AF regarding cardioembolic risk. AF is often asymptomatic (Hindricks et al., 2020), and long-term diagnostic tools such as the smartwatch (Perez et al., 2019) and internal loop recorder (Gladstone et al., 2015) are useful to diagnose subclinical AF. PACs as an AF predictor could be a marker of undiagnosed AF leading to ischemic stroke. However, an increased PAC burden is a predictor of ischemic stroke occurrence, independent of AF occurrence (Larsen et al., 2015). PACs can directly lead to abnormalities in atrial function. Patients with a high PAC burden seem to have reduced atrial function (O’Neal et al., 2016b), leading to a decrease in left atrial stroke volume and potential thrombus formation (Goyal and Spodick, 2001). Nonsustained atrial arrhythmia seems to be a clinical marker of atrial cardiomyopathy leading to an increased cardioembolic risk. The risk of ischemic stroke recurrence in ESUS patients with a CHA_2_DS_2_-VAS_c_ score > 2 is associated with increased PAC burden or AF (Larsen et al., 2015). PAC occurrence is associated with LA dilation and risk factors for atrial cardiomyopathy in an ESUS population (Pinho et al., 2015; Vinther et al., 2017; Cha et al., 2020), suggesting that PACs are a clinical marker of atrial cardiomyopathy (Sajeev et al., 2019). Furthermore, the presence of PACs after AF ablation results in a significant decrease in left atrial reverse remodeling (Hasegawa et al., 2019), highlighting a direct role of PACs in atrial cardiomyopathy.

## Current limitations and clinical implications

**FIGURE 2:**
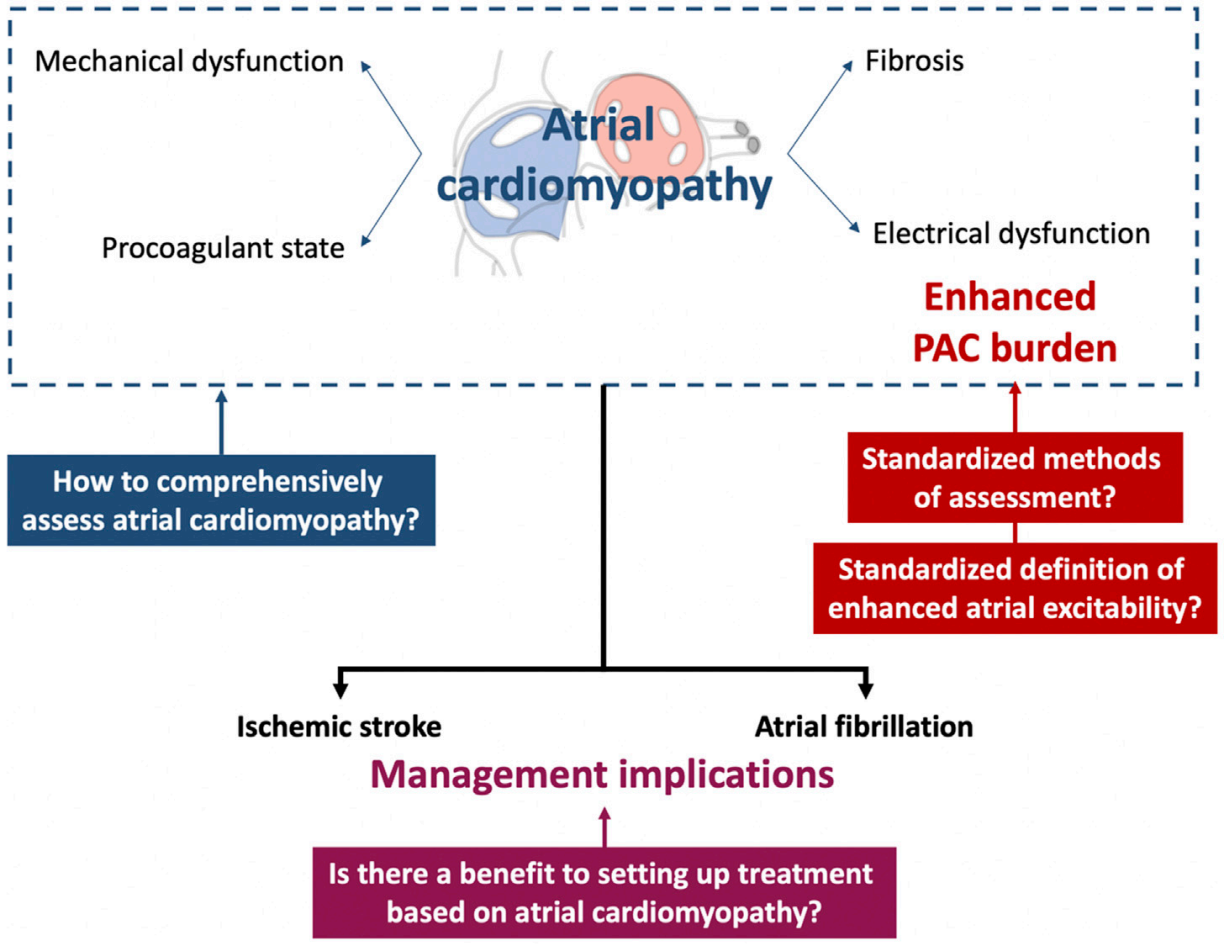
Current issues regarding the concept of nonsustained atrial arrhythmia as a marker of atrial cardiomyopathy and potential clinical implications. PAC, premature atrial contraction.

The relationship between nonsustained atrial tachyarrhythmias and atrial cardiomyopathy is supported by different epidemiological and clinical features. However, several issues remain unresolved regarding the detection of atrial cardiomyopathy and the contribution of the assessment of ectopic atrial rhythm on the one hand and the therapeutic management of atrial cardiomyopathy on the other hand (Figure 2).

The threshold of PAC burden predicting atrial cardiomyopathy has to be precised. Indeed, the cutoffs used in the different studies reported in this review are heterogeneous, e.g., at least one PAC per 24 h (O’Neal et al., 2016a), more than 72 PACs in 24 h (Lin et al., 2015), and more than 30 PACs per hour (Larsen et al., 2015), although the threshold of 500 PACs/24 h has been proposed in a consensus paper (Arnar et al., 2019). A standardized methodology for the evaluation of atrial hyperexcitability has not yet been validated. Different diagnostic tools are used in clinical practice, such as standard 12-lead ECG (Inohara et al., 2013; Qureshi et al., 2014; O’Neal et al., 2016a), 15-s (Murakoshi et al., 2015) to 2-min (Cheriyath et al., 2011) ECG, and 24-h (Engström et al., 2000; Acharya et al., 2015) to 48-h (Binici et al., 2010; Larsen et al., 2015) Holter monitoring. Screening for nonsustained atrial arrhythmia will need to be considered in light of the use of new diagnostic tools such as implantable loop recorders (Kochhäuser et al., 2014; Gladstone et al., 2015) and digital devices (Varma et al., 2021).

Standardized therapeutic management of nonsustained atrial arrhythmia is not currently consensual. Regarding a rhythm control strategy using beta-blockers, prospective interventional trials are lacking, and observational studies suggest a lack of benefit in terms of AF occurrence and ischemic stroke (Huang et al., 2022). Invasive PAC ablation is an effective strategy regarding the PAC burden (Wang et al., 2017), but its value in improving clinical prognosis has not been depicted. Regarding cardioembolic risk prevention, no randomized controlled trials (RCTs) have evaluated the benefit of anticoagulation in cases of a high PAC burden. It is not clear whether PACs directly lead to TER or are a marker of atrial cardiomyopathy. If the latter is true, therapeutic management of PACs would be unnecessary to reduce the occurrence of ischemic stroke (Marcus and Dewland, 2015; Alhede et al., 2017; Heijman et al., 2017).

Assessment of a high PAC burden could be a relevant diagnostic tool for the characterization of atrial cardiomyopathy but does not lead to the detection of structural and functional atrial abnormalities on its own: a combined diagnostic approach seems necessary. Other noninvasive electrophysiological tools, such as the P-wave terminal force in lead V_1_, the P-wave duration, the maximum P-wave area, and advanced interatrial block, could be used (Mączyńska et al., 2018) since they are strong predictors of AF occurrence and ischemic stroke occurrence in patients with ESUS and the general population (Kamel et al., 2015b, 2015a; He et al., 2017; Martínez-Sellés et al., 2020). Different biomarkers of atrial endothelial dysfunction, inflammation, coagulation, or myocardial stress also appear to be relevant diagnostic tools (Markus et al., 2021). Imaging could also play an essential role in assessing atrial morphological abnormalities using echocardiography (Xu et al., 2020) and assessments of structural abnormalities and fibrosis (Akoum et al., 2013).

The setting up of therapeutic strategies targeting atrial cardiomyopathy remains poorly studied. In AF patients, the current guidelines highlight the potential role of upstream therapies such as angiotensin-converting enzyme inhibitors, beta-blockers, and statins to prevent atrial remodeling as a rhythm control strategy (Hindricks et al., 2020). However, conflicting results among various studies limit the recommendation of upstream therapies for the general AF population. Cardioembolic risk prevention using anticoagulation in a population exhibiting atrial cardiomyopathy without AF has not been evaluated thus far (Bayés de Luna et al., 2017). The ongoing ARCADIA study is the first RCT evaluating the benefit of anticoagulation with respect to the occurrence of all-cause stroke in patients with a history of ESUS and atrial cardiomyopathy (Kamel et al., 2019). Atrial cardiomyopathy is not defined by a high PAC burden but using composite criteria, including increased P-wave terminal force in V1, NT-proBNP > 250 pg/ml, and left atrial dilation on an echocardiogram.

## Conclusion

Nonsustained atrial arrhythmia does not appear to be solely a predictor of AF but is also an independent predictor of ischemic stroke *via* the presence of atrial cardiomyopathy. However, the diagnostic and therapeutic management of a high PAC burden to prevent AF and ischemic stroke remains partially studied.
